# Supplementary material for: A Synthetic Polymicrobial Community Biofilm Model Demonstrates Spatial Partitioning, Tolerance to Antimicrobial Treatment, Reduced Metabolism, and Small Colony Variants Typical of Chronic Wound Biofilms
Source: Pathogens. 2023 Jan 10;12(1):118. doi: 10.3390/pathogens12010118 (PMC9862141; doi:10.3390/pathogens12010118)
Supplement: Supplementary file 1 [file pathogens-12-00118-s001.zip › pathogens-2127872-supplementary.pdf]

Supplementary materials

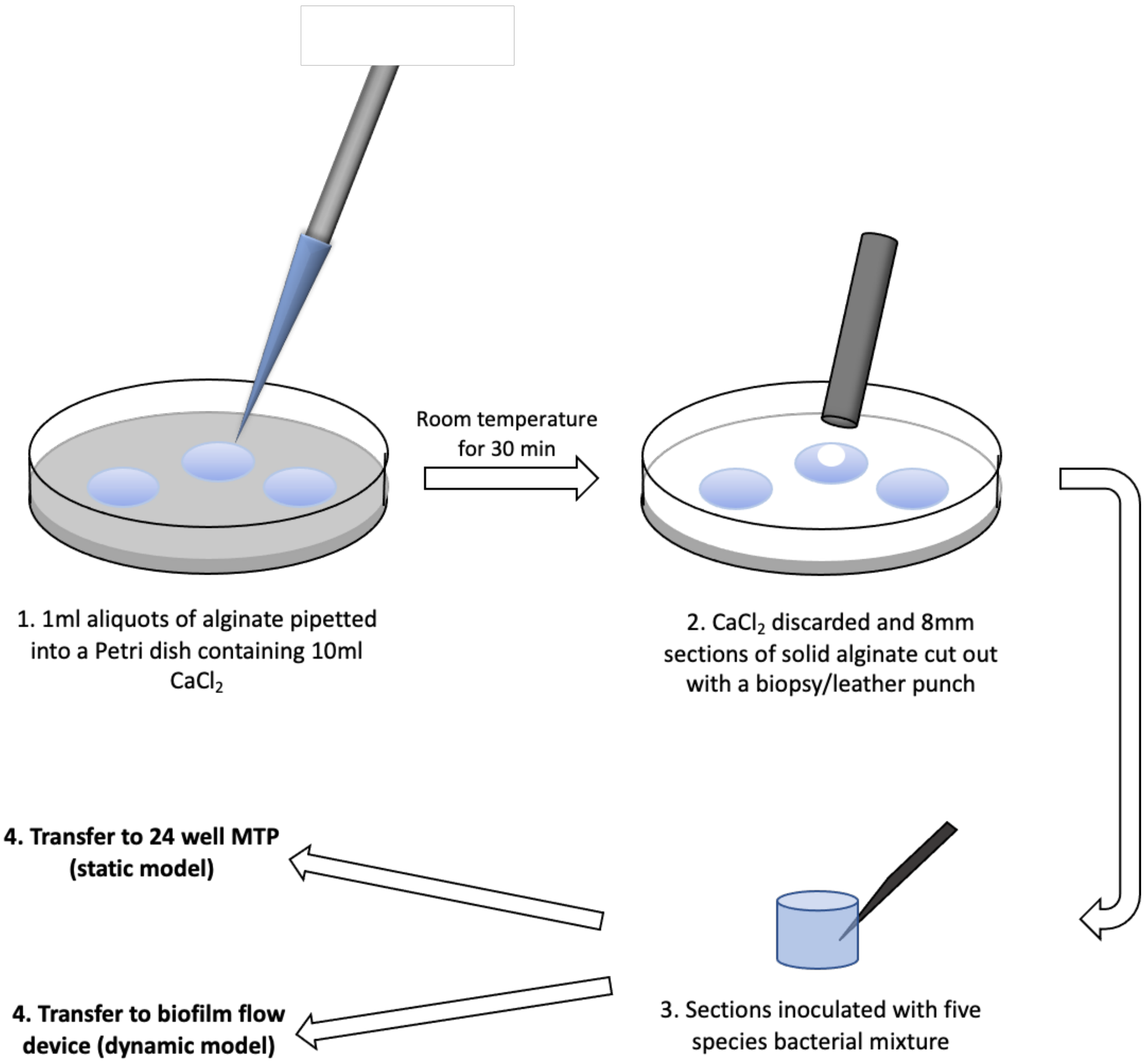

Figure S1. Schematic representation of biofilm preparation using an alginate matrix inoculated with bacterial suspension.

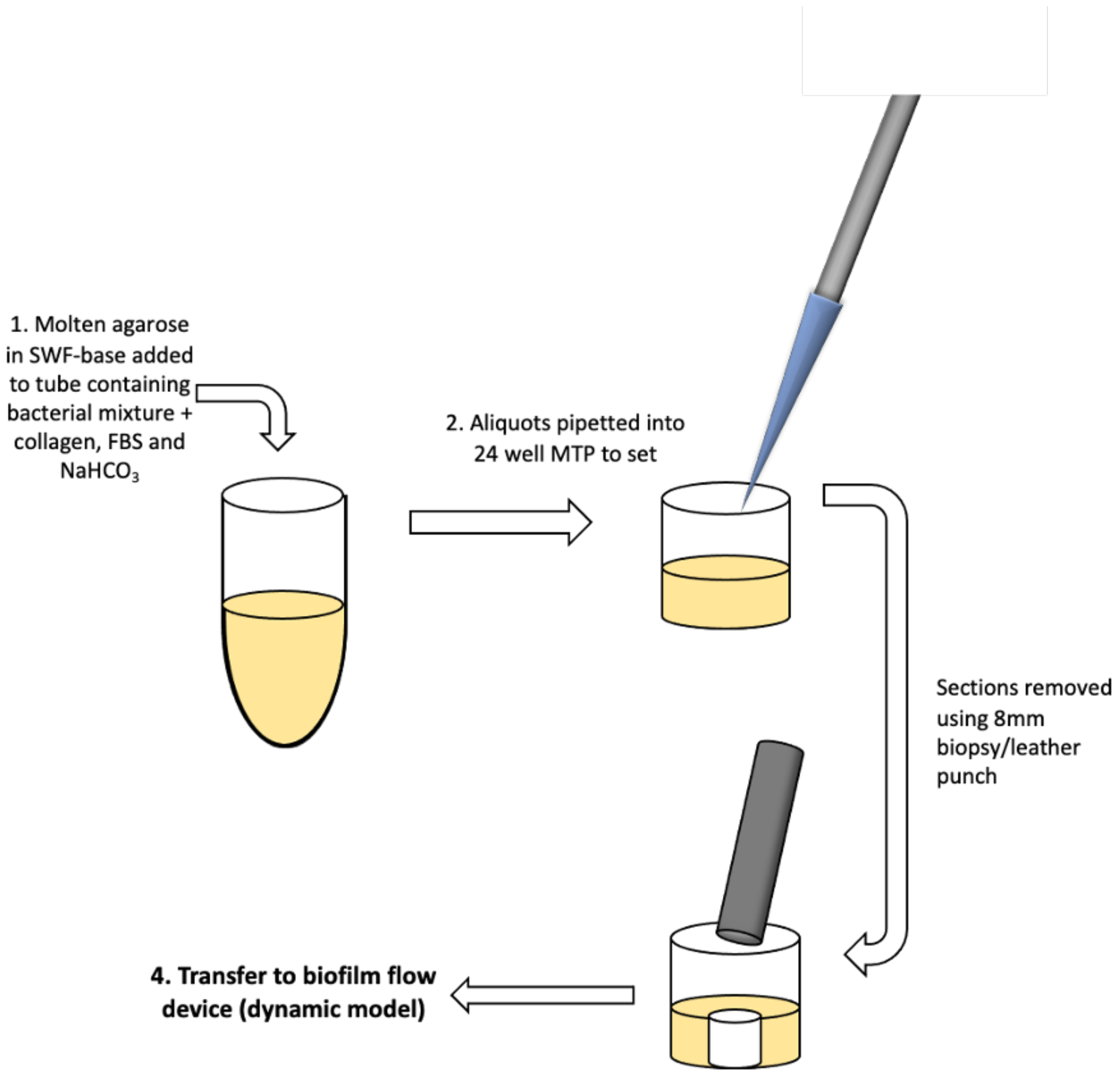

Figure S2. Schematic representation of biofilm preparation using an agarose-collagen matrix in which bacteria are embedded.

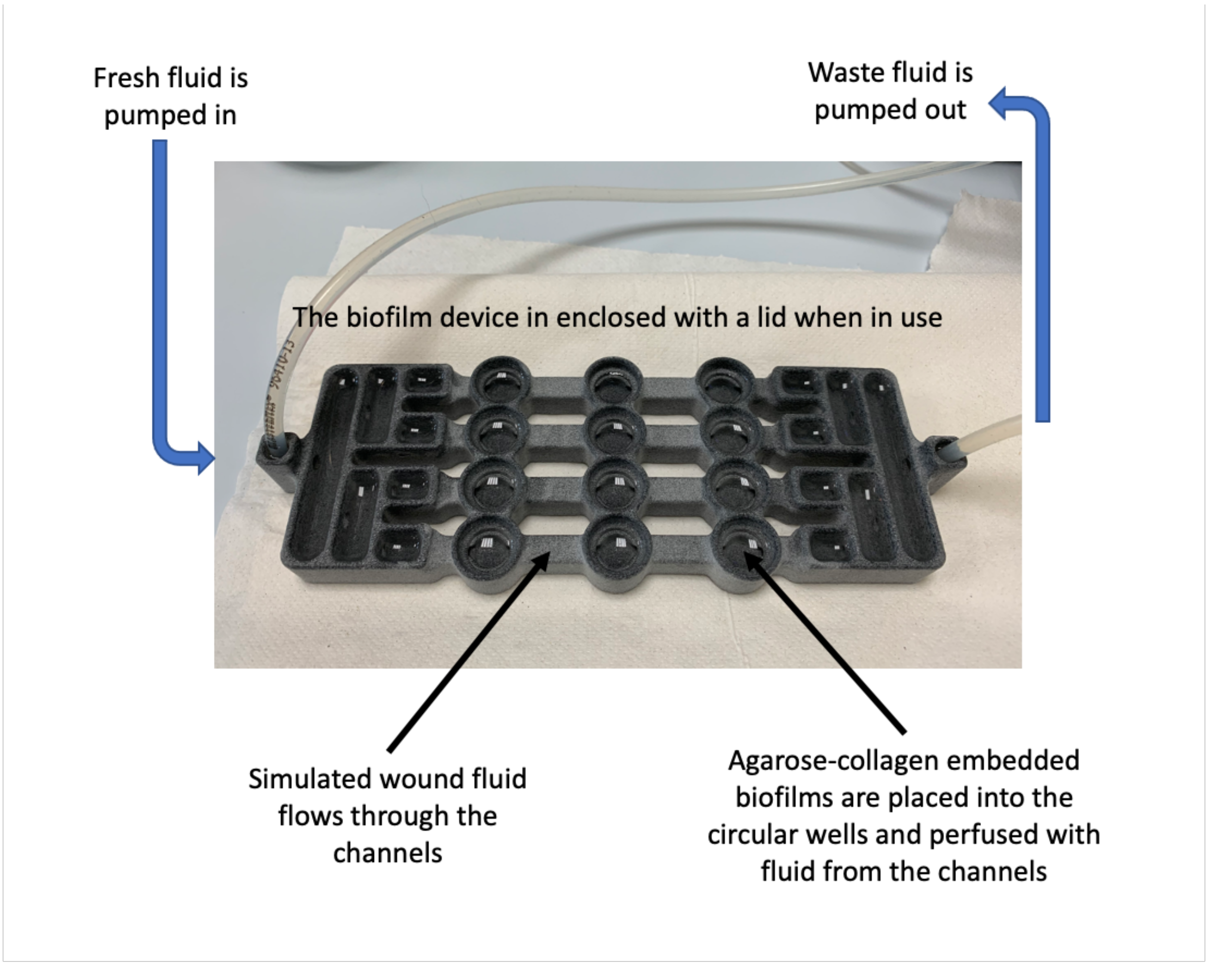

Figure S3. Biofilm flow device when prepared for mixed-species biofilm growth under flow, using biofilms embedded in an agarose-collagen matrix.
